# Novel use of calcium sulfate bioabsorbable beads as a cement restrictor in revision arthroplasty for infection

**DOI:** 10.1308/rcsann.2024.0119

**Published:** 2025-03-25

**Authors:** CJ Manning, H Wynn-Jones

**Affiliations:** Wrightington, Wigan and Leigh Teaching Hospitals NHS Foundation Trust, UK

## Background

Antibiotic-loaded bone cement is commonly used in single-stage revision hip and knee replacements for treating prosthetic joint infection (PJI). It serves to fix the implant and deliver high concentrations of antibiotics. Absorbable calcium sulfate beads (e.g. Stimulan®) are also used for antibiotic delivery. Cement restrictors occlude the medullary canal, aiding in pressurisation and preventing excess cement migration. We describe a novel use of antibiotic-loaded calcium sulfate beads as an absorbable cement restrictor.

## Technique

The primary treatment for any PJI is thorough debridement of all necrotic tissue and removal of all prosthetic material. After this, the bone and intramedullary canal are prepared for re-implantation of the revision prosthesis. In the case of cemented stemmed implants, it is necessary to occlude the canal of the femur and/or the tibia before cementation.

**Figure 1:**
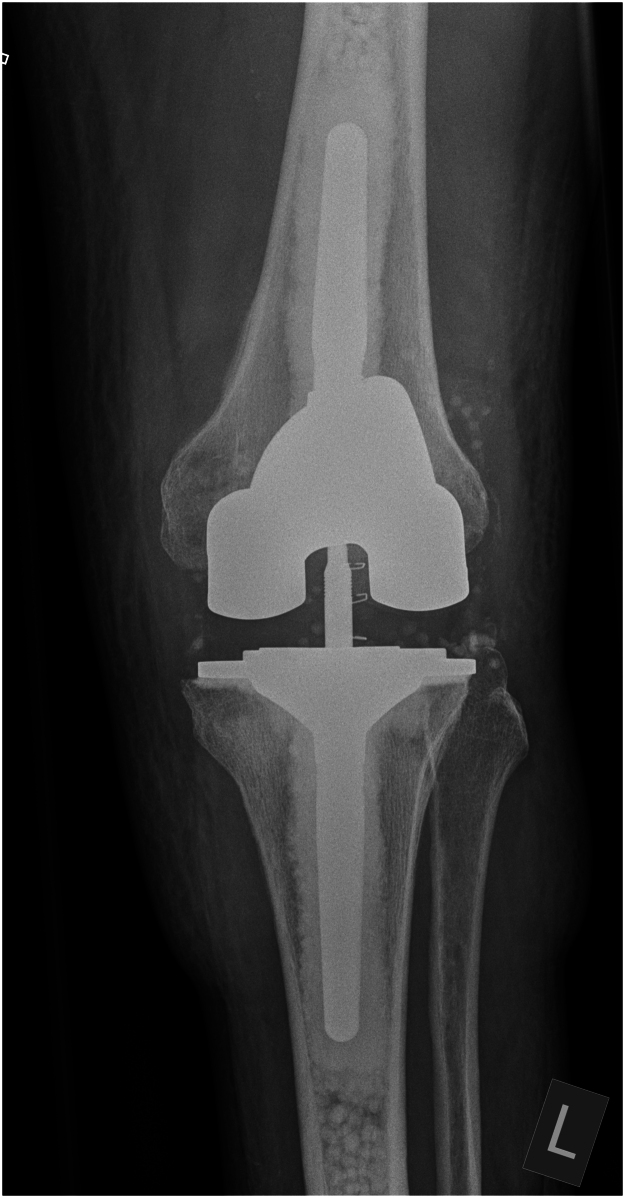
Revision total knee replacement immediately post surgery. The anteroposterior x-ray of the knee demonstrates uniformly distributed antibiotic-loaded calcium sulfate beads in the medullary canal. The beads are visible as small, discrete radio-opaque spheres in the distal femur and proximal tibia, proximal to the implant and cement mantle, indicating effective cement restriction.

**Figure 2:**
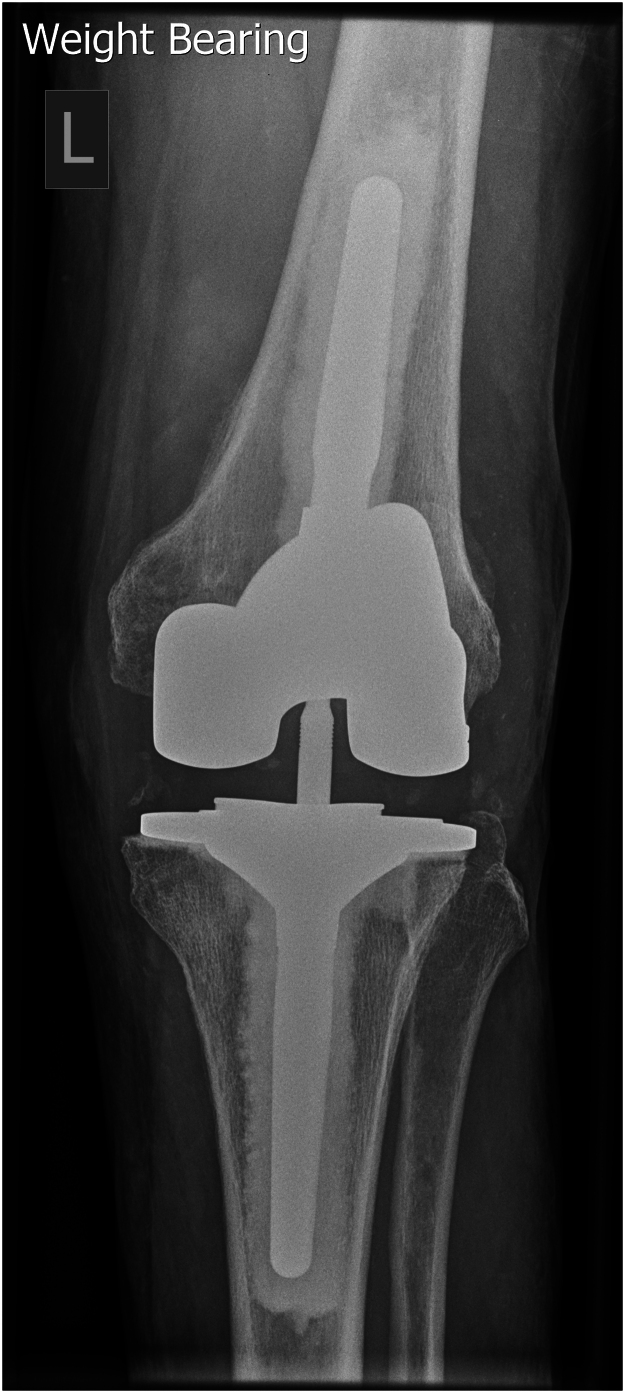
Revision total knee replacement 3 months post surgery. A postoperative x-ray taken 3 months after revision knee arthroplasty demonstrates absorption of the antibiotic-loaded calcium sulfate beads as a cement restrictor. The image shows that the cement mantle remains intact, indicating successful pressurisation and structural support without the retention of foreign material. This confirms the dual role of antibiotic-loaded calcium sulfate beads in both infection control and maintaining the integrity of the cement mantle.

**Figure 3:**
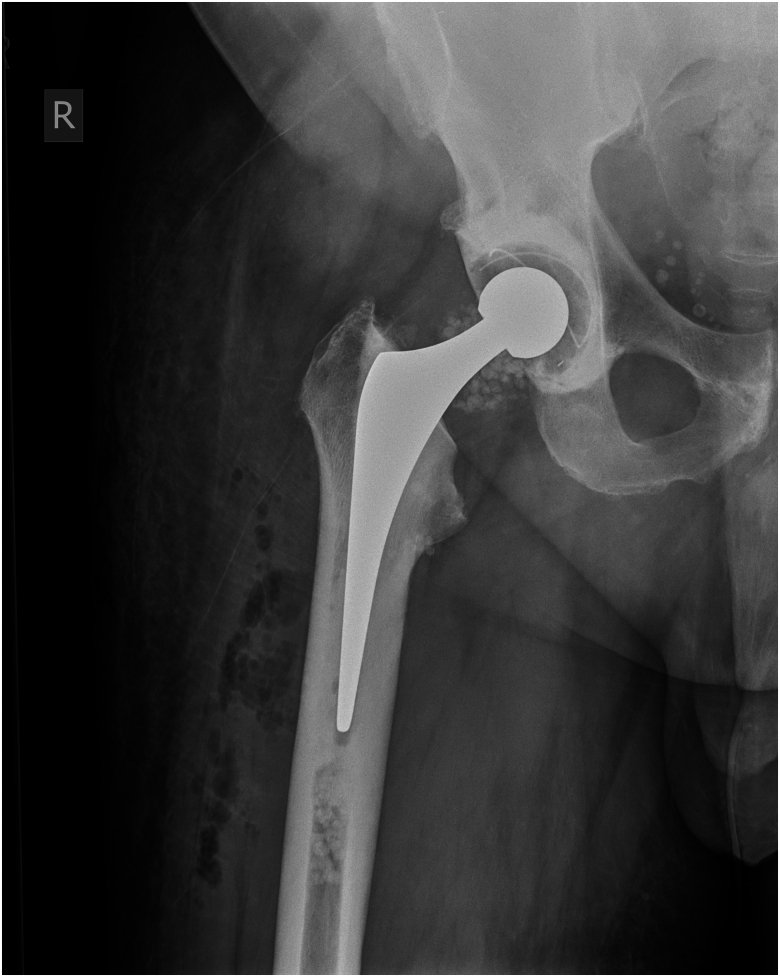
Revision total hip replacement immediately post surgery. The anteroposterior x-ray of the hip demonstrates the antibiotic-loaded calcium sulfate beads acting as an effective cement restrictor in the medullary canal.

**Figure 4:**
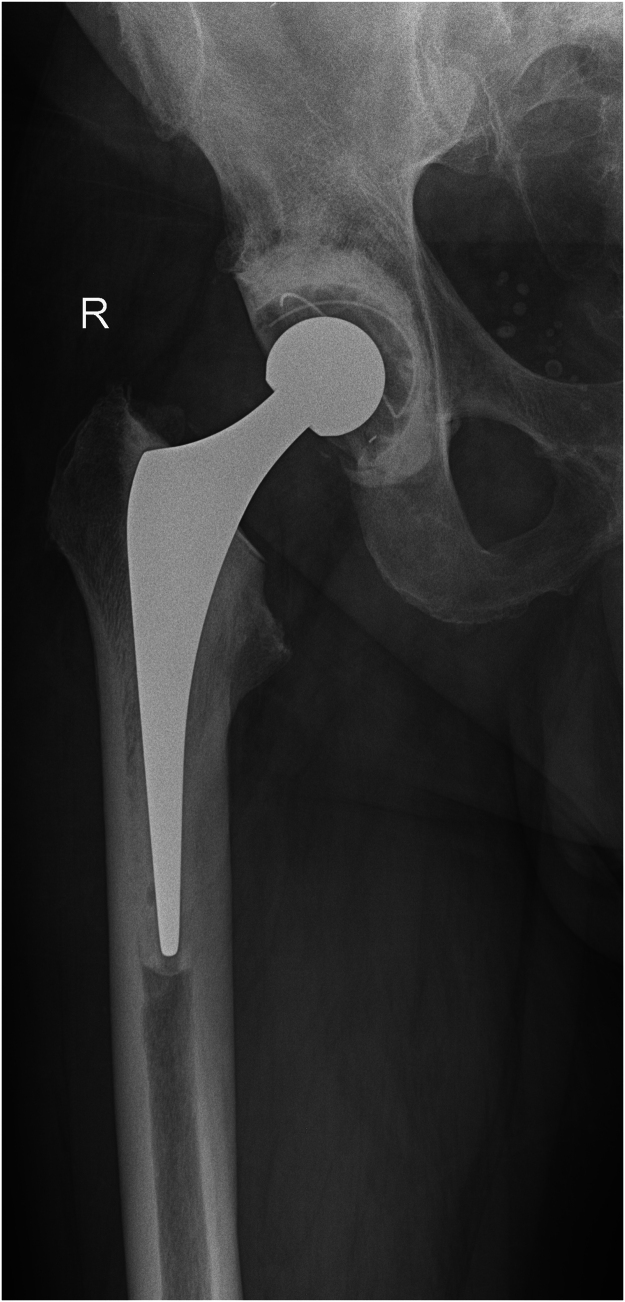
Revision total hip replacement 3 months post surgery. A postoperative x-ray taken 3 months after revision hip arthroplasty demonstrates absorption of the antibiotic-loaded calcium sulfate beads.

We used a combination of calcium sulfate bead sizes, impacted with generic orthopaedic tamps of appropriate diameter and length for the patient’s anatomy. The insertion depth is determined by the implant stem length and desired cement mantle. This technique was used in both femoral and tibial canals for a revision knee replacement (Figures 1 and 2) and in the femoral canal for a revision hip replacement (Figures 3 and 4). X-rays in Figures 1 and 3 confirm canal occlusion and appropriate cement pressurisation, and after 3 months, Figures 2 and 4 show resorption of the beads and maintenance of the cement mantle.

## Discussion and Conclusion

This technique extends the already established use of antibiotic-loaded calcium sulfate beads for the treatment of PJI. This technique produces a very effective cement restrictor that releases antibiotics into the canal, minimises the length of the prosthetic construct and is bioabsorbable.

